# From correlation to causation: analysis of metabolomics data using systems biology approaches

**DOI:** 10.1007/s11306-018-1335-y

**Published:** 2018-02-27

**Authors:** Antonio Rosato, Leonardo Tenori, Marta Cascante, Pedro Ramon De Atauri Carulla, Vitor A. P. Martins dos Santos, Edoardo Saccenti

**Affiliations:** 1 Magnetic Resonance Center and Department of Chemistry “Ugo Schiff”, University of Florence, Florence, Italy; 2 Department of Experimental and Clinical Medicine, University of Florence, Florence, Italy; 3 CIBER de Enfermedades hepáticas y digestivas (CIBERHD, Madrid) and Department of Biochemistry and Molecular Biomedicine, Universitat de Barcelona, Barcelona, Spain; 4 Laboratory of Systems and Synthetic Biology, Wageningen University & Research, Wageningen, The Netherlands; 5 LifeGlimmer GmbH, Berlin, Germany

**Keywords:** Pathway, Network analysis, Correlation network, Association network, Enrichment analysis

## Abstract

**Introduction:**

Metabolomics is a well-established tool in systems biology, especially in the top–down approach. Metabolomics experiments often results in discovery studies that provide intriguing biological hypotheses but rarely offer mechanistic explanation of such findings. In this light, the interpretation of metabolomics data can be boosted by deploying systems biology approaches.

**Objectives:**

This review aims to provide an overview of systems biology approaches that are relevant to metabolomics and to discuss some successful applications of these methods.

**Methods:**

We review the most recent applications of systems biology tools in the field of metabolomics, such as network inference and analysis, metabolic modelling and pathways analysis.

**Results:**

We offer an ample overview of systems biology tools that can be applied to address metabolomics problems. The characteristics and application results of these tools are discussed also in a comparative manner.

**Conclusions:**

Systems biology-enhanced analysis of metabolomics data can provide insights into the molecular mechanisms originating the observed metabolic profiles and enhance the scientific impact of metabolomics studies.

## Introduction

The pioneering experimental work of Mamer and Horning (Horning and Horning 1971; Mamer and Crawhall 1971) and the first application by Pauling (1971) laid the bases for metabolomic profiling of samples. These approaches constituted the precursors of today’s metabolomics techniques. It was with the work of Oliver (1998) and Trethewey (1999) that metabolomics established itself as a standalone discipline and then became a core component of systems biology (SB), providing an integrated view of biochemistry in complex organisms (Nicholson and Lindon 2008). The rapid evolution and spreading of metabolomics leveraged the technical developments of Nuclear Magnetic Resonance (NMR) and Mass Spectroscopy (MS), which made metabolomics experiments widely accessible.

**Fig. 1:**
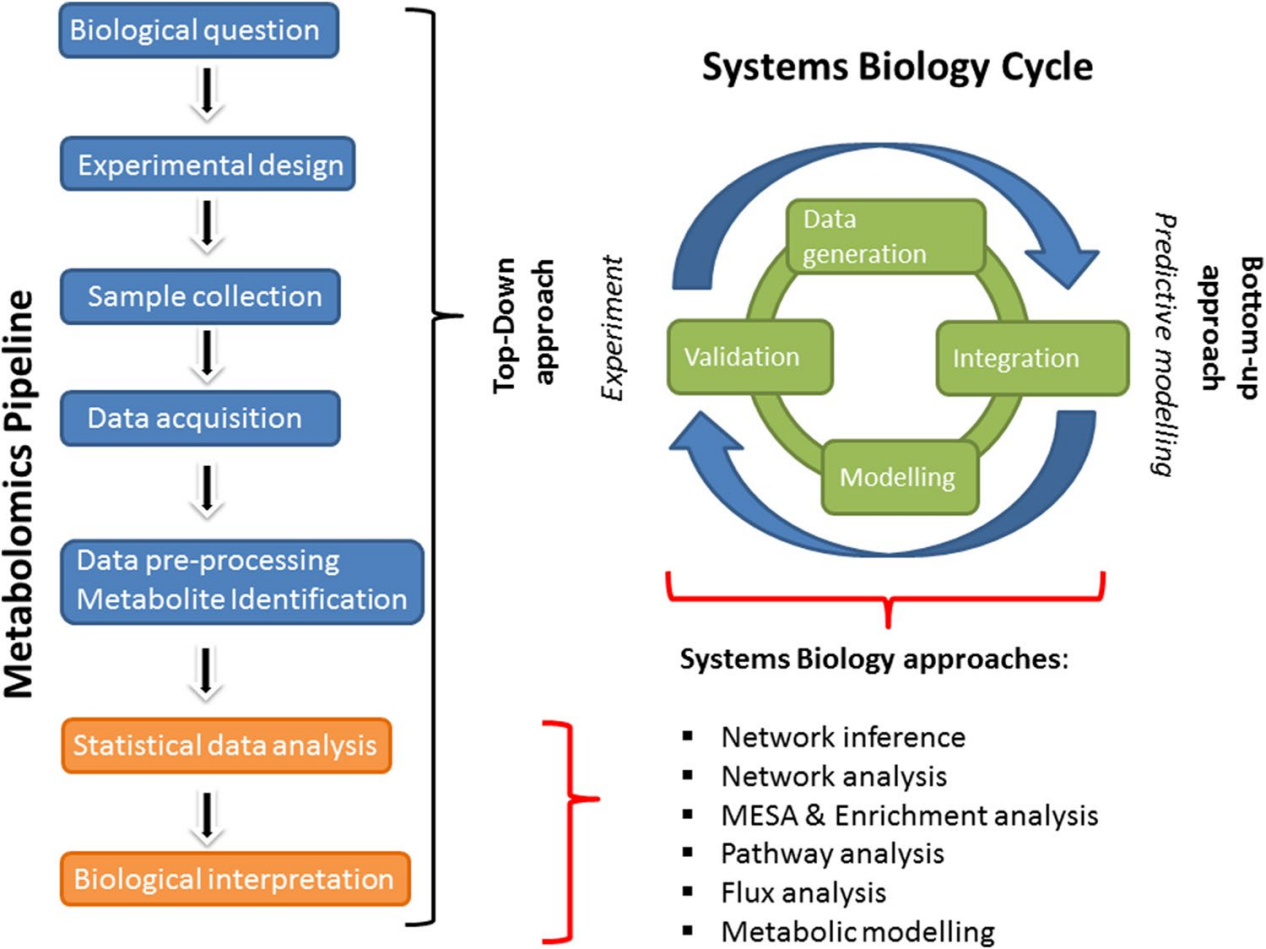
Relationship between the systems biology cycle and the metabolomics pipeline

In the top-down approach of SB (see Fig. 1), hypotheses about the regulatory mechanisms are drawn upon the analysis of patterns observed in metabolite profiles. Such hypotheses can be tested in new experiments in an iterative cycle (Bruggeman and Westerhoff 2007). In fact, metabolomics takes a special position among the *omics* disciplines in the SB top–down approach: the metabolome is the endpoint of biological processes, carrying imprints of genetic, epigenetic and environmental factors, and thus it can provide the link between genotype and phenotype (Fiehn 2002; Griffin 2006; Krumsiek et al. 2016). A crucial demonstration of this concept was the observation that metabolomics measurements can reveal phenotypes for proteins active in metabolic regulation, even if their deletion does not change metabolic fluxes, such as growth rate (Raamsdonk et al. 2001).

Contextually with experimental advancements, researchers soon realized that the potential of metabolomics data could be exploited by deploying multivariate and pattern-recognition methods. The use of components methods, such as principal component analysis and factor analysis was established early (Meuzelaar and Kistemaker 1973; Windig et al. 1980). Then, metabolomics became rapidly intertwined in an almost symbiotic fashion with chemometrics (Trygg et al. 2007; van der Greef and Smilde 2005; Wishart 2007). This alliance has resulted in the development of a vast array of different tools for extracting (bio)chemically relevant information from measured (bio)chemical data, representing and displaying such information, and getting it into databases (Wold 1995; Wold and Sjöström 1998; Spicer et al. 2017).

Chemometrics proved to be pivotal in studies that showcased the potential of metabolomics (Assfalg et al. 2008; Holmes et al. 2008; Nicholson et al. 2011). However, nowadays data analysis based on chemometrics alone may be considered the major bottleneck for further advancement of metabolomics itself. Chemometrics approaches have an intrinsic exploratory nature, and thus their application to metabolomics analyses typically generates novel biological hypotheses that need validation. Moving from research generating hypotheses towards research generating mechanistic insight about biological problems would constitute a major advance for the omics fields (Yates 2016). One way to achieve this is to deploy systems biology approaches, such as network analysis and metabolic modelling, to investigate metabolomics data. This may open new avenues to obtain biological knowledge from transcriptomics, proteomics and metabolomics studies and will allow researchers to leverage all omics to contextualize their results.

In line with the concepts outlined above, in this review we did not cover the approaches to data analysis that are typical of chemometrics and statistical analysis, such as supervised and regression methods (e.g., Partial Least Square Discriminant Analysis, principal component regression) or unsupervised tools (e.g., Principal Component Analysis, cluster analysis). Instead, we focused on systems biology approaches like network inference and metabolic modelling.

## Metabolite identification and mapping

An important aspect underlying most if not all the methods for the analysis of metabolomics data that we will address in the next sections is to properly identify the metabolites in the MS or NMR spectra and map them within the metabolic context of the organism. Often the peaks detected in experiments are assigned based on reference spectra contained in large chemical database. However, the analytical methods used in metabolomics do not allow coverage of the whole range of small molecules produced by an organism, introducing possible biases in the interpretation of whole-organism metabolism. Although this is a very broad theme, in this section we will try to summarize the features of some tools for metabolite mapping that can be exploited in the context of systems biology approaches.

Metabolome Searcher (Dhanasekaran et al. 2015) is a web-based application (http://procyc.westcent.usu.edu/cgi-bin/MetaboSearcher.cgi) to directly search genome-constructed metabolic databases. Its aim is to enhance the identification of MS data by using compound databases derived empirically. Incorporating information on genome-encoded metabolism facilitates the identification of MS peaks that may not be present in standard chemical databases. Only the compounds that the organism of interest is able to produce, based on its genome, are investigated for potential matches. The output metabolites are mapped also to known metabolic pathways.

The MassTRIX web server (Suhre and Schmitt-Kopplin 2008) (http://masstrix3.helmholtz-muenchen.de/masstrix3/) addresses the annotation of putative metabolites by providing a hypothesis-driven approach to interpret MS data. MassTRIX processes the submitted list of raw mass peaks by comparing the input experimental masses against all chemical compounds of the Kyoto Encyclopedia of Genes and Genomes (KEGG) database (Kanehisa et al. 2015, 2017), additionally including ^13^C, ^15^N and other isotopes and optionally adding selected lipids. Then it presents the identified chemical compounds in their genomic context as differentially coloured objects on KEGG pathway maps. By adding transcriptomics data or information on differences in the gene complement (e.g. samples from different bacterial strains), the user can interpret the metabolic state of the organism in the context of its actual or potential enzymatic capacities.

A similar approach was also employed in MetaMapp (Barupal et al. 2012). MetaMapp is a tool to integrate biochemical pathways (using the KEGG reactant pair database) and chemical relationships (using the Tanimoto chemical similarity score and the mass spectral similarity score of the National Institute of Standards and Technology, NIST) to map the metabolites detected in MS and/or NMR experiments in a network graph. Such graphs can be displayed in Cytoscape (Shannon et al. 2003). MetaMapp is independent of the experimental technology utilized to identify metabolomics profiles, thus providing a way to integrate and visualize data from different metabolomics platforms.

MetExplore (Cottret et al. 2010) is a computational pipeline designed to map chemical libraries on genome-scale metabolic networks. This tool can be used to obtain statistics on the experimental coverage of organism-specific metabolic networks. The main purpose of MetExplore is to provide an interactive visualization of metabolic networks (or sub-networks) to mine metabolomics (and other “omics”) data. After the mapping is performed, MetExplore permits to visualize metabolites in the context of the whole network, a specific pathway, a selection of pathways or a selection of reactions.

Another recent tool integrating automated analysis of mass spectrometry data and visualization of biological context by linking each metabolite to one or more biological pathways (see also next section) is the Polyomics integrated Metabolomics Pipeline (PiMP) (Gloaguen et al. 2017). This tool annotates metabolites identified in mass spectrometry experiments, providing direct access to the experimental features supporting each annotation, and then allows users to jump directly to the pathway(s) relevant for each metabolite. However, this is a visualization tool and does not perform pathway analysis as described in Sect. 6.

Pre-existing biochemical knowledge about metabolic pathways may provide useful information for the assignment of unknown compounds in large metabolomics datasets. Gipson et al. (2008) exploited this idea by developing a computational protocol to improve UPLS-MS metabolite assignment through the matching of peak correlation pairs (from acquired MS data) with a database of biochemically relevant interaction pairs (pathway data from the KEEG database). A stochastic local search optimization algorithm was implemented to select the putative peak assignment that maximizes both the correlations and the strength of correlations in each cluster of MS peaks, in agreement with the most likely metabolic pathway from the database.

Integrated approaches that combine transcriptome, proteome and metabolome profiling have gained popularity and have proven to provide novel insights in the understanding of the biological systems (Cho et al. 2008; Jiang et al. 2015; Kolbe et al. 2006). A first approach to the interpretation of complex omics experiments is the joined visualization of the data on templates that collect previous knowledge. In this frame, the Paintomics web server (http://www.paintomics.org) (García-Alcalde et al. 2011) provides a simple but effective resource for integrated visualization in studies where transcriptomics and metabolomics data are generated on the same set of samples. The inputs to the server are gene expression and metabolite quantifications, which are then displayed on KEGG maps.

The web-based ProMeTra system (Neuweger et al. 2009) (https://omictools.com/prometra-tool) allows users to combine datasets from heterogeneous multiple-omics sources. This tool visualizes and combines datasets from transcriptomics, proteomics, and metabolomics on user defined metabolic pathway maps. ProMeTra supports pathway maps designed and annotated by the users.

There are only a few tools explicitly devoted to the analysis of metabolomics data. Metscape (Gao et al. 2010) (metscape.ncibi.org) is a plug-in for Cytoscape (Shannon et al. 2003), developed to visualize and interpret metabolomics data in the context of human metabolic networks. Metscape allows users to trace the connections between metabolites and genes, visualize compound networks and display compound structures as well as information for reactions, enzymes, genes, and pathways. Experimental data can be visualized and explored as networks and as a function of time or experimental conditions. A subsequent redesign of Metscape (Metscape 2) (Karnovsky et al. 2012) allows users to enter experimental data and display them in the context of relevant metabolic networks to identify enriched pathways from expression profiling data.

Table 1 presents a list of the tools for mapping metabolites into biochemical pathways mentioned in this section.

**Table 1:** Tools for mapping metabolites into biochemical pathways

Name	Description	Reference	URL
NA	Refine mass assignments through the intersection of peak correlation pairs with a database of biochemically relevant interaction pairs	Gipson et al. (2008)	NA
Metabolome Searcher	Simplify database search in MS databases by limiting the query to genome plausible metabolites	Dhanasekaran et al. (2015)	http://procyc.westcent.usu.edu/cgi-bin/MetaboSearcher.cgi
MassTRIX	Presents the MS identified chemical compounds in their genomic context as differentially coloured objects on KEGG pathway maps	Suhre and Schmitt-Kopplin (2008)	http://masstrix3.helmholtz-muenchen.de/masstrix3/
MetaMapp	Map the detected metabolites in a MS experiment in a network graph	Barupal et al. (2012)	NA
MetExplore	To provide an interactive visualization of metabolic networks (or sub-networks) to mine metabolomics data	Cottret et al. (2010)	http://metexplore.toulouse.inra.fr/joomla3/index.php
Paintomics	Provide a simple but effective resource for integrated visualization in studies where transcriptomics and metabolomics data are generated on the same set of samples	García-Alcalde et al. (2011)	http://www.paintomics.org
KaPPa-View	A web-based tool for representing quantitative data for individual transcripts and/or metabolites on plant metabolic pathway maps	Tokimatsu et al. (2005)	http://kpv.kazusa.or.jp/
MapMan	A user-driven tool that displays large data sets onto diagrams of metabolic pathways or other processes	Thimm et al. (2004)	http://mapman.gabipd.org/web/guest
ProMeTra	Visualizes and combines datasets from transcriptomics, proteomics, and metabolomics on user defined metabolic pathway maps, with the ability to generate enriched SVG images or animations via a user-friendly web interface	Neuweger et al. (2009)	https://omictools.com/prometra-tool
Metscape	Allows users to trace the connections between metabolites and genes, visualize compound networks and display compound structures as well as information for reactions, enzymes, genes, and pathways	Gao et al. (2010)	http://metscape.ncibi.org/

## Analysis of metabolomics data using network approaches

The most natural extension and complementation of methods based on covariance/correlation for the analysis of multivariate metabolomics data [such as principal component analysis or covariance simultaneous component analysis (Smilde et al. 2015)] is their representation and analysis as networks. Networks constitute a powerful view to understand biological systems where not only the individual components are considered, but also their interconnections and their function as a whole (Ma’ayan 2011; Weckwerth and Fiehn 2002).

A biological network is a graphic representation of objects (called nodes) and their relationships (described by links or edges). It can be conveniently described using a matrix, termed adjacency or connectivity matrix **A**. The rows and columns of **A** represent the nodes, i.e. metabolite concentrations or abundances. Here, we refer generically to metabolite concentration. Strictly speaking, this is correct only for targeted metabolomics experiments where the concentrations of metabolites are determined using appropriate standards. In general, MS experiments provide metabolite abundances, which can be considered a proxy for concentrations, whereas NMR provides quantities in arbitrary units that are proportional to concentrations. However, from a numerical point of view this is not relevant for the computational methods presented here, but it might be relevant for the biological interpretation of the data. The non-zero elements of **A** are real numbers that describe the strength of the relationship between any two nodes. The relationship between two metabolites can be very diverse in nature: for instance, one can postulate the existence of such relationship if their concentration levels are highly correlated, if they participate in the same metabolic pathway, or if they are directly connected through some biochemical reaction. Within this context, it should be noted that metabolomics data can be used to reconstruct metabolic networks at different levels (topology, stoichiometry, directionality and kinetics) using dedicated experiments. In this review, we focus on the application of network approaches to analyze metabolomics data that usually have not been gathered with the aim of reconstructing entire metabolic networks. For the latter purpose, the typical starting point is genome data (see also some of the tools mentioned in the previous section and in Table 1). Nevertheless, some approaches are available to build genome-scale metabolic networks from raw high resolution mass spectroscopy data (Jourdan et al. 2007; Moritz et al. 2017). Methods to reconstruct metabolic networks have been reviewed elsewhere (Frainay and Jourdan 2017; Hendrickx 2013; Hendrickx et al. 2011).

Table 2 presents a list of network-based methods applicable to metabolomics studies. These methods are discussed in the following sections.

**Table 2:** List of network inference methods used in metabolomics studies

Acronym	Name	Reference
ARACNE	Algorithm for the reconstruction of accurate cellular networks	Margolin et al. (2006)
CLR	Context likelihood of relatedness algorithm	Faith et al. (2007)
CORR	Correlation	
PCLRC	Probabilistic context likelihood of relatedness of correlation algorithm	Saccenti et al. (2014)
PIUmet	Prize-collecting Steiner forest algorithm for integrative analysis of untargeted metabolomics	Pirhaji et al. (2016b)
WCGNA	Weighted correlation gene network analysis	Zhang and Horvath (2005)

### Association networks

The nodes in a network are associated (connected) based on some similarity measure: in metabolomics the similarity between metabolites, and thus their association, is usually expressed using Pearson or Spearman’s correlation indexes. Consequently, the elements of the corresponding adjacency matrix are in the interval [−1, 1] (Cakır et al. 2009). This kind of networks is sometimes called correlation or relevance networks. Biological information can be derived considering both the magnitude and the sign of correlations: for instance, strong positive correlation ($\left| \rho \right|>0.9$) between two metabolites can indicate a condition of rapid equilibrium or enzyme dominance, while strong negative correlation can indicate the presence of a conserved moiety (Camacho et al. 2005). In general, the correlations observed in metabolomics data are the result of the combination of all reactions and regulatory processes in the network (Hendrickx 2013; Stelling et al. 2004; Steuer et al. 2003). Surprisingly, there may be no correlation between metabolites that are close in a metabolic pathway. For instance, in wild type potato tubers, glutamate and glutamine are metabolic neighbors in the glutamine synthase pathway, but appear to be uncorrelated (*ρ* = 0.0243, Spearman). Instead, valine and methionine are strongly correlated (*ρ* = 0.951) even if they are not metabolic neighbors (Camacho et al. 2005; Weckwerth et al. 2004). The information encoded in the correlation matrix may be not fully sufficient to reverse engineer the underlying enzymatic system (Steuer et al. 2003). Still, it can be used as a proxy to describe a given physiological state of the system of interest, as the correlation matrix can change with the steady-state concentrations of metabolites (Fukushima et al. 2011). It is then reasonable to assume that differences or communalities in the biological processes are reflected in the characteristics of the inferred correlation networks (Szymanski et al. 2009). This is the rationale for the use of association networks to analyse metabolomics data.

The zero elements of the adjacency matrix can be selected based on the statistical significance of the pairwise metabolite correlations. This was the approach used in (Ursem et al. 2008), one of the first papers to deploy a network approach to the analysis of metabolomics data, where Pearson correlations were calculated among pairs of metabolites measured using gas chromatography–mass spectrometry (GC–MS) in tomato samples. The advantage over principal component analysis (PCA) is that network plots do not focus on the representation of maximum variation in data matrices, which may negatively affects data interpretation. Indeed, the relationships between metabolites whose variation is spread out over several principal axes can be easily overlooked in PCA biplots (Ursem et al. 2008). The work of Ursem et al. (2008) built on previous works, where correlation analysis was used to unravel molecular mechanisms (Kose et al. 2001; Roessner et al. 2001; Steuer et al. 2003; Urbanczyk-Wochniak et al. 2003).

Yang et al. (2012) performed a correlation network analysis on urine metabolomics data from patients suffering of central precocious puberty taking a hybrid approach. First, they identified metabolites discriminating between cases and controls using a Partial Least Squares (PLS) approach and then mapped them on a reconstruction of a global human metabolic network using the KEGG database (Kanehisa et al. 2015, 2017). The discriminating metabolites had significantly higher degree, betweeness and closeness than the global network.

Another commonly used approach is to binarize the adjacency matrix by imposing a threshold *τ* for the correlation | *ρ*| between any pair of metabolites and/or a threshold α on the associated *P*-value. This is usually called hard thresholding, as exemplified below:

1 $$A_{{ij}}^{{}} \to \left\{ {\begin{array}{*{20}{c}} {1{\text{ if }}\left| {{\rho _{ij}}} \right|>\tau {\text{ }}({\text{and }}P<\alpha )} \\ {\,\,\,\,\,\,\,\,0{\text{ otherwise }}} \end{array}} \right.$$

The choice of the threshold *τ* is fundamental since it ultimately drives the topology of the resulting networks. In an analysis of tissue- and/or genotype-dependent metabolomics correlations in Arabidopsis, Fukushima et al. investigated the effect of varying the correlation threshold and found that the number of groups of connected metabolites showed a transition from small to large at *τ* = 0.5, which they subsequently used (Fukushima et al. 2011). They commented that such a threshold does not guarantee explicit biological significance. However, this value is not far from 0.6, which was indicated as a lower bound for low/weak correlations in metabolomics data (Camacho et al. 2005) and used by other authors (Ghini et al. 2015; Saccenti et al. 2016; Suarez-Diez and Saccenti 2015). Szymanski et al. (2009) applied a threshold α = 0.01 on the *P*-value of the correlation after Bonferroni correction for multiple testing and used bootstrapping to obtain robust correlation estimation.

The patterns of correlations between metabolites can be compared across different conditions to identify associations that are disrupted or altered by pathophysiological conditions with respect to a healthy or control status, an approach referred to as differential network analysis. Hu et al. (2015) addressed the problem of finding disrupted connections in osteoarthritis by taking a statistical approach that exploited a permutation test to assess the significance of changes in the correlations of two metabolites across different conditions. Similarly, Szymanski et al. (2009) considered metabolite correlation networks from Escherichia coli exposed to different environmental stress conditions and compared network characteristics to pinpoint possible mechanisms underlying stress response.

**Fig. 2:**
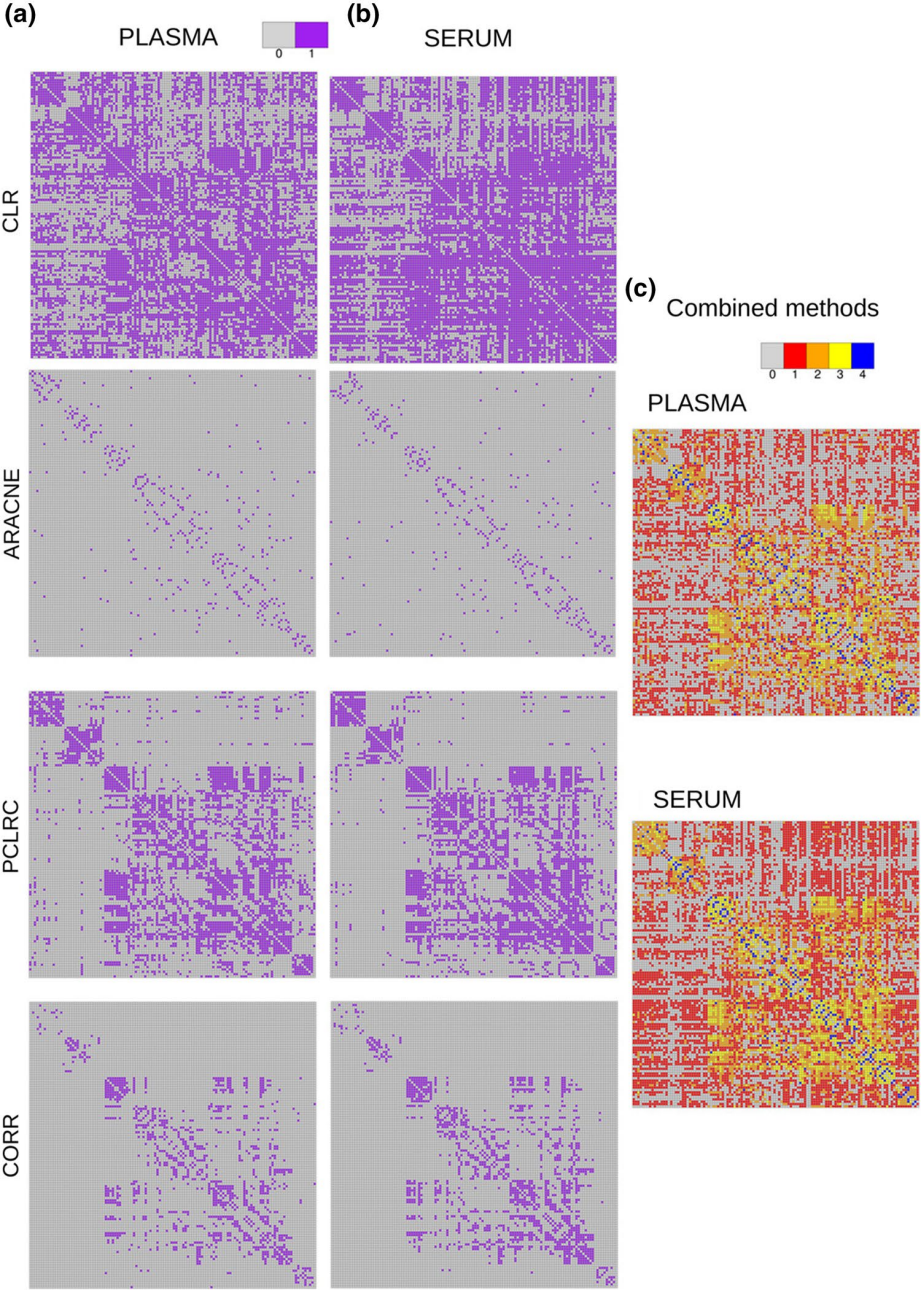
Association network of 133 blood metabolites measured using MS/MS on 2139 subjects. **a** Plasma metabolites association networks obtained using the four different methods. **b** Serum metabolites association networks obtained using the four different methods. **c** Consensus association network for serum and plasma. *CLR* context likelihood of relatedness, *ARACNE* algorithm for the reconstruction of accurate cellular networks, *PCLRC* probabilistic context likelihood of relatedness on correlations, *CORR* Pearson’s correlation). Reproduced with permission from Suarez-Diez et al. (2017). Copyright (2017) American Chemical Society

Saccenti et al. (2014) investigated the latent cardiovascular risk of healthy subjects by considering highly connected metabolites, the so called *hubs*, and reported differential behaviour of Very Low Density Lipoprotein (VLDL) and glucose in high and low risk cardiovascular risk networks. They applied a combined method, by analysing association networks with a multivariate approach to highlight differences among networks pertaining to different risk phenotypes (see Fig. 2). Hubs are nodes that are much more connected than average or typical nodes, and consequently are very likely to play crucial biological roles. The concept of hubs was first introduced within the analysis of yeast protein–protein interaction networks (Jeong et al. 2001).

The correlations observed for metabolomics data are usually small ($\left| \rho \right|<0.6$) because of the systemic nature of metabolic control. As previously mentioned, two metabolites can be poorly correlated even if they are neighbours in a metabolic pathway because the variance in the enzymes that control them can affect their levels to the same extent and in different directions (Camacho et al. 2005). Metabolites are generated through fast biochemical reactions in an open mass-flow system. Consequently, they can be considered to be in a quasi-steady state when compared to the time scales of the upstream regulatory processes. This results also in indirect, system-wide correlations between distantly connected metabolites (Lee et al. 2008). The latter phenomenon can be taken into account using partial correlations, i.e. considering pairwise correlation between two variables with the effect of a set of controlling random variables removed. Krumsiek et al. (2011) used Gaussian graphical models, a type of undirected network representation where the relationships among metabolites are expressed as partial correlations, to analyse a large human population cohort. They found this approach to generate more sparse and robust networks with modular structure than those based on Pearson’s correlations, and observed that high partial correlation coefficients generally correspond to known metabolic reactions. This is a striking result since associations in a correlation networks do not necessarily correspond to and/or represent metabolic reactions (Marcotte 2001; Steuer et al. 2003). Using the same approach, Krumsiek et al. (2015) investigated sex-related differences in metabolite association networks and found several submodules across different pathways that were strongly gender-regulated.

As a word of caution, it is important to consider that the results of network inference (and data analysis in general) can be affected by data pre-treatment (also known as pre-processing) such as scaling, transformation and normalization. Such pre-treatments are routinely applied to metabolomics data in order to correct for systematic and unwanted variation such as sample-to sample to variability induced by dilution effects (e.g. in the case of urine) or differences in experimental settings (like different sample titration or different number of scans in NMR experiments). The literature on the topic is huge: we refer the reader to Bijlsma et al. (2006), Goodacre et al. (2007), Saccenti (2016), Van Den Berg et al. (2006) and references therein for more information.

### Weighted correlation networks

Weighted gene correlation network analysis (WCGNA) is a systems biology method for describing the correlation patterns among genes across microarray samples. WCGNA can be used for finding clusters (modules) of highly correlated genes, for summarizing such clusters using the so-called module eigengene, i.e. a representative gene summarizing the expression profile of the module (Langfelder and Horvath 2007), or an intramodular hub gene, for relating modules to one another and to external sample traits (Langfelder and Horvath 2008). When applied to metabolite profiles rather than to gene expression profiles WCGNA can be considered an extension of correlation network inference. While correlation networks are based on the similarity of metabolites profiles as expressed by the correlation coefficients, WCGNA in based on the dissimilarity profiles obtained from the so-called topological overlap matrix (TOM). Using the TOM makes the networks less sensitive to spurious connections or to connections missing due to random noise (Ravasz et al. 2002; Zhao et al. 2010). However, also the TOM is based on the correlation between any pair of metabolites; indeed, the element *w*_*ij*_ of TOM is defined as

2 $${w_{ij}}=\frac{{{l_{ij}}+{a_{ij}}}}{{\hbox{min} ({k_i},{k_j})+1 - {a_{ij}}}}$$

where

3 $${a_{ij}}=corr{({m_i},{m_j})^\beta }$$

4 $${l_{ij}}=\sum\limits_{u} {{a_{iu}}{a_{uj}}}$$

and *m*_*i*_ and *m*_*j*_ denote metabolite *i*-th and *j*-th, *k*_*i*_ and *k*_*j*_ denote the connectivity of metabolite *i*-th and *j*-th. The dissimilarity is defined as 1 − *w*_*ij*_, which is a measure of interaction between metabolites weighted by the strength of their correlation. The parameter $\beta$ is chosen to achieve a scale-free topology and its choice is a fundamental step in WCGNA. Clusters of metabolites are obtained by applying a hierarchical clustering algorithm on the dissimilarity matrix in order to assign the metabolites to different modules based on a dynamic branch height cutting algorithm (Langfelder et al. 2007).

DiLeo et al. (2011) applied WCGNA to NMR metabolomics data collected from developmentally staged tomato fruits belonging to several genotypes. With this approach, they could recognize and model systems-level differences in biological networks even where the poorly defined phenotypes precluded the use of PCA or other multivariate approaches.

Lusczek et al. (2013) applied WCGNA to investigate pathophysiologic state associated with traumatic injury and haemorrhagic shock through the analysis of scale-invariant metabolic network which were constructed from NMR urinary metabolic profiles. They could define network modules (i.e. clusters of functionally related metabolites) related, for examples, to tricarboxylic acid (TCA) cycle or to aerobic metabolism. Within those modules they identified hub metabolites related to cellular respiration, highlighting its fundamental role in the pathophysiology of haemorrhagic shock and to late resuscitation time points. They observed that PLS discriminant analysis (PLS-DA) did not capture the significance of several hub metabolites, which emerged only in the network analysis. In the same work (Lusczek et al. 2013), the authors discussed also the limitation of the WCGNA approach. Such limitations rest on the assumptions that the network shows a scale-free topology, that is with few metabolites highly connected and many metabolites with low connectivity; this translates in the connectivity *P*(k) and the clustering coefficient *C(k)* to follow a power law. The authors found *P(k)* to follow a power law but not *C(k)*, indicating the absence of modular structure in the network of urinary metabolites. They suggested that this may be caused by *(i*) urine being a waste product in which little to no active metabolism occurs and *(ii*) the limited number of metabolites considered (n = 60) which is less than the content of the full urinary metabolome. A further hypothesis put forward in the same work was that networks constructed from metabolite profiles derived from biological samples that are metabolically active, such as blood or tissue, may exhibit power law (i.e. a few metabolites connected with many metabolites) behaviour in both connectivity and clustering coefficients. However, in contrast to gene regulatory network, expression networks or metabolic networks, the metabolite correlation networks have not been fully characterized in terms of network topology (i.e. the patterns of interconnection among the nodes). Therefore, it is not very clear what are the expected or more likely network properties (e.g. small-world networks, distribution networks). We refer the reader to (Lee et al. 2008; Nikiforova et al. 2005; Weckwerth et al. 2004) and references therein for more on this topic.

### Approaches from functional genomics

Since one of the major challenges in systems biology is the reconstruction of gene regulatory networks, many methods have been developed for this scope (Marbach et al. 2012) and some of them have been deployed in metabolomics. Saccenti et al. (Suarez-Diez and Saccenti 2015) compared two methods for the inference of regulatory networks, ARACNE (Algorithm for the Reconstruction of Accurate Cellular Networks) and PCLR (Probabilistic Context Likelihood of Relatedness Algorithm), to reconstruct blood metabolite association networks. Both these methods leverage mutual information. Given two discrete variables *A* and *B* (describing, for instance, metabolite concentrations), the mutual information *MI(A,B)* between *A* and *B* is defined as

5 $$MI(A,B)=\sum\limits_{{i,j}}^{n} {p({a_i},{b_j})\log \frac{{{\text{p}}({a_i},{{\text{b}}_j})}}{{{\text{p}}({a_i}){\text{p}}({b_j})}}}$$

where *p(a*_*i*_,*b*_*j*_*)* is the joint probability distribution function of *A* and *B*, and *p(a*_*i*_*)* (respectively *p(b*_*j*_*))* indicates the probability that *A* = *a*_*i*_ (respectively *B* = *b*_*j*_). It should be noted that the mutual information between two variables is not independent from correlations, since, under some conditions, the two variables can be functionally related (Song et al. 2012). The following sections describe the two approaches in some detail.

#### The algorithm for the reconstruction of Accurate cellular networks (ARACNE)

ARACNE (Algorithm for the Reconstruction of Accurate Cellular Networks) (Margolin et al. 2006) assigns to each pair of metabolites an association weight equal to their mutual information. It then takes into account triplets of edges connecting metabolites *i, j* and *k* in the network. The weakest association of each triplet is considered to be indirect (spurious) and pruned, i.e. set to 0, if the difference between the two lowest weights is above a cut-off value *ξ*. In practice, the following two conditions are evaluated for each triplet *i, j, k*:

6 $$\left\{ \begin{gathered} MI(i,j)<MI(j,k) - \xi \hfill \\ MI(i,j)<MI(i,k) - \xi \hfill \\ \end{gathered} \right.$$

The weighted adjacency matrix is transformed into a binary topological matrix by additionally imposing a threshold on the mutual information. The threshold is usually 0, leading to all non-zero values being transformed to 1. Saccenti et al. (2015) observed that ARACNE produces extremely sparse metabolites association networks; nevertheless, most of the associations deemed relevant by the ARACNE algorithm were also recovered by the other algorithms assessed in the study, indicating that it was able to reconstruct the backbone of the association network.

#### The context Likelihood of relatedness (CLR) algorithm

The CLR algorithm (Faith et al. 2007) estimates the likelihood of the mutual information *MI(i, j)* between two metabolites by defining a null model that considers all the possible MI values [*MI*_*i*_] and [*MI*_*j*_] for metabolites *i* and *j*. The following equations define the likelihood *f*

7 $$f({z_i},{z_j})=\sqrt {z_{i}^{2}+z_{j}^{2}}$$

where

8 $${z_i}=\hbox{max} \left\{ {0,\frac{{M{I_i}(i,j) - {\mu _i}}}{{{\sigma _i}}}} \right\}$$

and *µ*_*i*_ and *σ*_*i*_ are, respectively, the mean and the standard deviation of the distribution of the [*MI*_*i*_] values: a weighted adjacency matrix is built with entries *f(z*_*i*_, *z*_*j*_*)*.

#### The probabilistic context likelihood of relatedness of correlation algorithm (PCLRC)

Saccenti et al. (2014) developed a novel version of the CLR approach by substituting the mutual information with correlation and using a resampling approach for robust inference of the correlations. In this implementation, two-thirds of the data are used to iteratively estimate pairwise correlations among metabolites retaining only the 30% strongest.

At each iteration a matrix **A**^it^ is built in such a way that $A_{{ij}}^{{it}}$ = 1 if there is an association between metabolites *i* and *j* and 0 otherwise; this procedure is repeated *K* times and the final weighted association network is constructed by averaging the entries of **A**^it^ over the *K* iterations. The weights constitute a probabilistic measurement of edge likeliness on which a threshold can be applied to obtain a binarized association network. This algorithm was used to construct association networks of blood metabolites characteristics of low and high latent cardiovascular risk (Saccenti et al. 2014; Zhao et al. 2010).

#### The wisdom of crowd approach

Saccenti et al. (2016) proposed a wisdom of crowd approach (Marbach et al. 2012) to define urine metabolite association networks in healthy subjects by considering the consensus obtained from four different approaches (ARACNE, CLR, PCLR and Pearson’s correlations) and deeming relevant only associations inferred by three or more methods. They modelled the subject-specific networks through a statistical mechanics approach (Menichetti et al. 2015), by defining a core network of metabolite–metabolite associations conserved across 31 subjects.

**Fig. 3:**
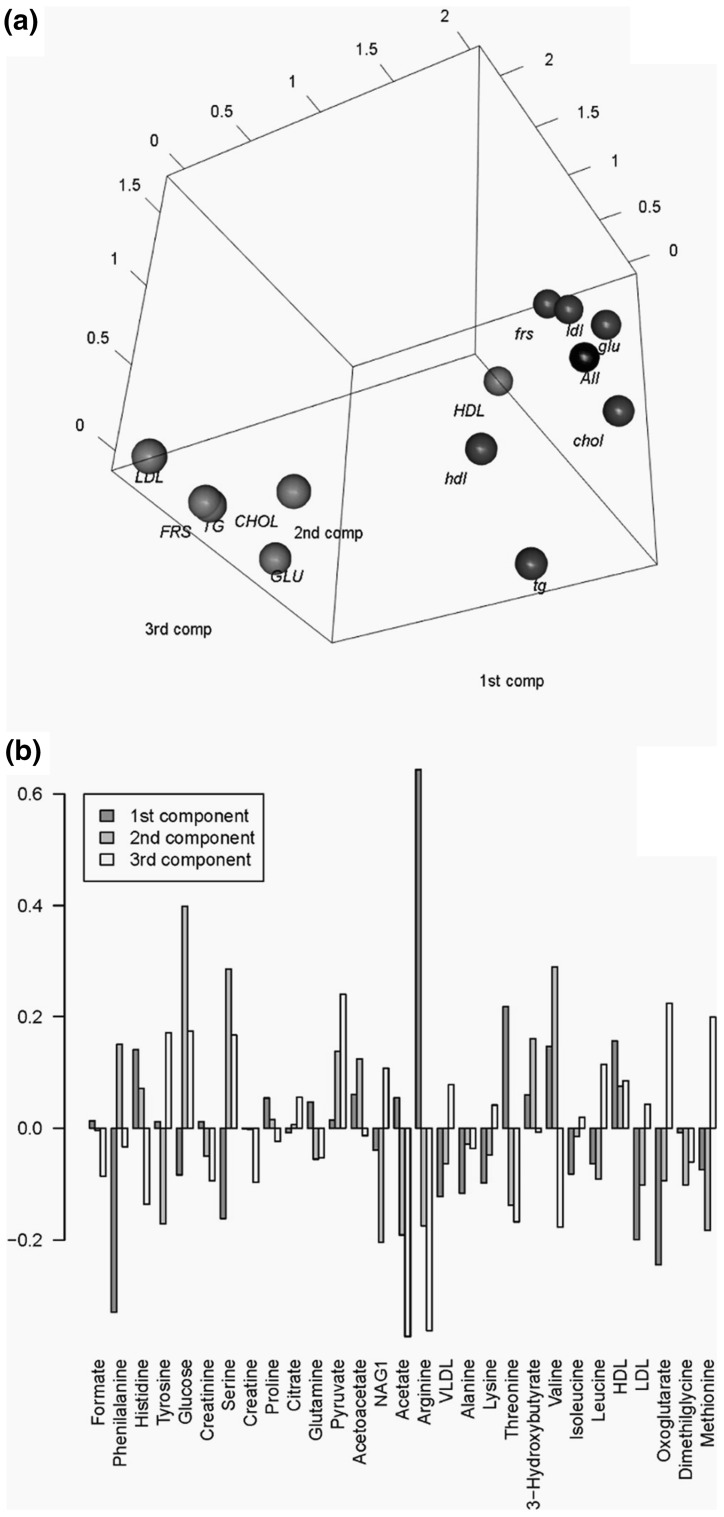
**a** Weight plot and **b** loadings plot of the INDSCAL model for the metabolite correlation network obtained using the *PCLCR* method. Each dot represents a network that corresponds to a given cardiovascular (CVD) risk parameter. Blue dots indicate low latent CVD risk, while red indicate high latent CVD risk. The associated CVD risk parameters are indicated in upper case for high risk and lower case for low risk. A reference network (indicated as “All”, black ball), built using all the subjects in the study, is given as reference. Reproduced with permission from Saccenti et al. (2014). Copyright (2014) American Chemical Society

The same approach was used in a study aiming to compare metabolite association networks obtained from serum and plasma samples. The networks were found to be topologically similar but showed local differences as in the case of amino acids (see Fig. 3) (Suarez-Diez et al. 2017). Similarly, Vignoli et al. (2017) studied sex- and age-specific association networks for metabolites in the plasma of healthy subjects. In particular, they investigated the different patterns of interconnectedness and observed sex-related variability in several metabolic pathways (branched-chain amino acids, ketone bodies and propanoate metabolism) as well as reduction in the connectivity of metabolite hubs linked to age in both sex groups.

#### Other methods

Pirhaij et al. (2016a) used their algorithm PIUmet (http://fraenkel-nsf.csbi.mit.edu/PIUMet/) to analyse and interpret untargeted liquid chromatography–mass spectrometry (LC–MS) data from lipidomics and phosphoproteomics experiments in a cell-line model of Huntington’s disease. Grounding on database information, the algorithm infers the identity of unassigned metabolites corresponding to features and the molecular mechanisms underlying their dysregulation. This innovative approach helps to reduce the bias towards well-studied metabolites typical of targeted metabolomics. The algorithm takes as input a list of LC–MS peaks that differ between two different conditions and searches for them in a databases containing over 42,000 nodes (either proteins or metabolites) connected by over one million weighted edges representing interactions between proteins as well as enzymatic and transporter reactions. The output is a subnetwork of the database representing metabolic pathways that are dysregulated under the conditions considered.

## Kinetic models

The metabolism is a network structure that can be approached as a system of interdependent variables that enable mathematical modelling through kinetic models. These models are defined as systems of ordinary differential equations describing the time course of metabolite concentrations as a function of rate laws that account for enzyme catalysis. The development of these models requires to know both the network structure and the reaction kinetics and parameters (Klipp et al. 2004). On the one hand, there is a large accumulated knowledge regarding the network structure, which is stored in databases like KEGG (Kanehisa et al. 2012), MetaCyc (Caspi et al. 2016) or Biomodels (Chelliah et al. 2015). Although this is a well-studied cellular level, the true structures can be importantly affected by factors like compartmentalization (de Mas et al. 2011; Nicolae et al. 2014) enzyme complexes and metabolic channelling (Castellana et al. 2014; Ovadi 1991). On the other hand, regarding reaction kinetics, there is also an accumulated knowledge, which can be explored in databases such as BRENDA (Scheer et al. 2011; Schomburg et al. 2013) or SABIO-RK (Wittig et al. 2012).

However, the details on enzyme kinetic parameters are available only for a minor part of the latter reactions (Büchel et al. 2013). In addition, the available measurements of the kinetic properties of enzymes historically come from systems reconstituted in vitro using purified enzymes (Savageau 1992). In this setting, the ideal conditions of homogeneity and free diffusion are fulfilled, and consequently the resulting models may neglect some factors affecting the kinetic properties, such as molecular crowding (Schnell and Turner 2004) and limited diffusion (Alekseev et al. 2016). To overcome these limitations, alternative approaches combine sampling methods with the integration of systemic available data and in vivo observations (fluxes, concentrations, perturbation experiments, …) (Andreozzi et al. 2016; Saa and Nielsen 2016; Stanford et al. 2013).

Alternative approaches take advantage of the current availability of data regarding the network structure and of the lineal nature of the system used to describe it, to apply optimization techniques to infer flux distributions (Fouladiha and Marashi 2017). Genome—scale models accounting for thousands of reactions are currently available (Chelliah et al. 2015; King et al. 2016; Swainston et al. 2016).

For those models including only the network structure as well as for complete kinetic models, it is useful to adopt techniques based on stable isotopes to know about the internal distribution of the metabolism. These are addressed in the next section.

## Metabolic flux modelling using stable isotope resolved metabolomics data

Although the analysis of metabolite correlative networks may not grasp the complete underlying metabolic mechanisms, it is certainly a valuable tool for the exploration of metabolomics data, as shown by the budding literature on the topic. The use of stable isotopes can provide a greater insight on the mechanisms that underlie the observed metabolomics profiles, permitting a direct analysis of mechanistic changes in metabolism. Each chemical reaction or transport process involved in a metabolic pathway is associated with a rate (flux) of transformation or transport. Mechanistic changes at the level of the metabolism are likely to produce changes in the distribution of fluxes. Intracellular fluxes are not directly measurable, but the use of stable isotope-enriched nutrients, such as 1,2-^13^C_2_-glucose or ^13^C_5_,^15^N_2_-glutamine, in in cell culture media and the application of Stable Isotope Resolved Metabolomics (SIRM) (Fan et al. 2012; Higashi et al. 2014) provides clues about the redistribution of carbon atoms along metabolic pathways. This can be used to estimate information about fluxes, such as their relative or absolute magnitudes (Lee 2006; Zamboni et al. 2005).

The estimation of fluxes based on the measured patterns of stable isotope labeling (especially using ^13^C) relies upon a combination of different methods, going from the direct interpretation of the labeling patterns to computational model-based approaches (Buescher et al. 2015; Niedenführ et al. 2015). Frequently, direct interpretation of labeling patterns is sufficient to provide information on the relative activities of pathways, on qualitative changes in pathway contributions via alternative metabolic routes, and on nutrient contribution to the production of different metabolites (Buescher et al. 2015). A recent example is the direct interpretation of the contributions of isotopic labeling tracers like 1,2-^13^C_2_-glucose to the synthesys of pentoses phosphate (Dong et al. 2017). The entry of this tracer into the oxidative pentose phosphate pathway results in the loss of the ^13^C tracer in position 1 in 1,2-^13^C_2_-glucose, contributing to the synthesis of ribose phosphate molecules that contain only one ^13^C atom (usually named M+1 pool of ribose-5-phosphate). Instead, the entry into the non-oxidative pentose phosphate pathway results in the synthesis of ribose phosphate molecules that contain two ^13^C atoms (usually named M+2 pool of ribose-5-phosphate). The subsequent entry of M+1 pentose-phosphate into glycolysis contributes to the synthesis of triose phosphate and lactate molecules with one ^13^C atom (M+1). An approximate estimation of the relative importance of oxidative versus non-oxidative pentose phosphate pathway fluxes can be inferred from the M+1/M+2 ratio of the RNA-derived ribose. During the last years, the use of this and other isotopic labelling tracers have been applied to unveil the different metabolic pathways activated in cancer cells (see for a review Dong et al. 2017).

**Fig. 4:**
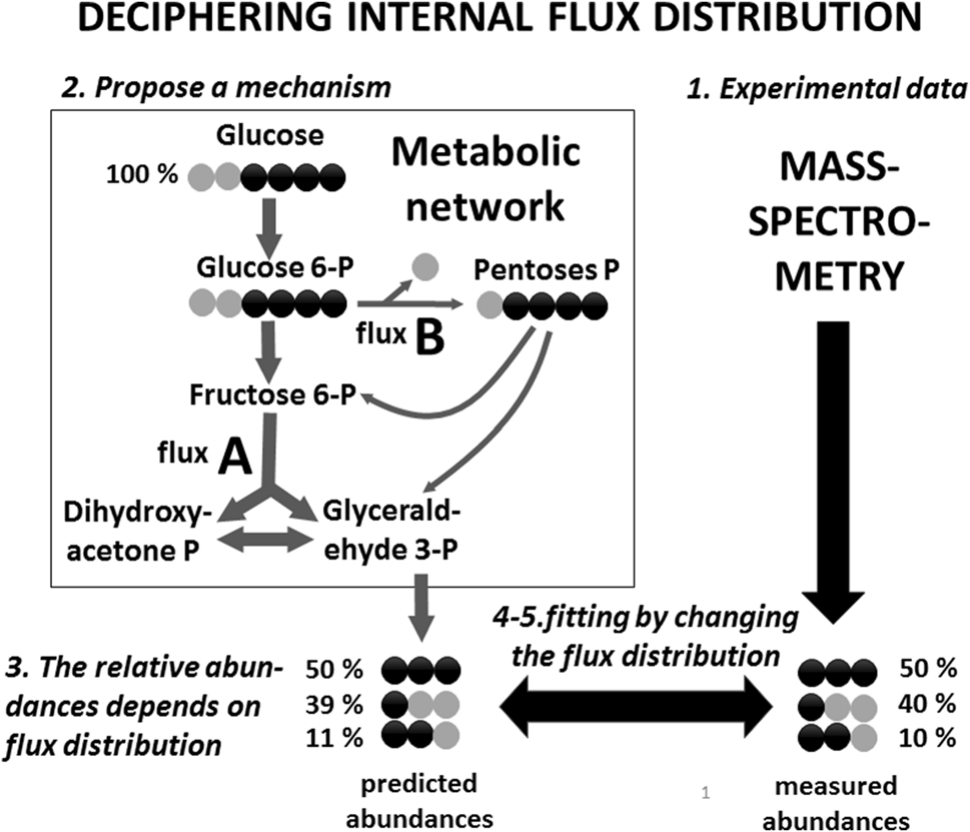
Overview of metabolic flux modelling using stable isotope resolved metabolomics data

By using computational approaches, all internal metabolic fluxes can be estimated simultaneously by combining the measured labeling patterns resulting from isotope propagation with the measured cellular uptake and secretion rates (Buescher et al. 2015). A reliable model of the relevant network of biochemical reactions is an indispensable input to the computational approach. The reliability of hypotheses regarding flux distributions can be evaluated by comparing measured and predicted isotopologue distributions. (Fig. 4). A variety of different methods are available (Crown and Antoniewicz 2013; Kruger and Ratcliffe 2009; Niedenführ et al. 2015; Sauer 2006; Wiechert and Nöh 2013; Zamboni 2011), together with specific software platforms: FiatFlux (Zamboni et al. 2005); Isodyn (Selivanov et al. 2005); METRAN (Yoo et al. 2008); OpenFlux (Quek et al. 2009); Influx_s (Sokol et al. 2012); 13CFLUX2 (Weitzel et al. 2013); INCA (Young 2014); WUFlux (He et al. 2016). In many cases, a system of balance equations around isotopomers—which depend on specific fluxes—is solved to predict label enrichments. Fluxes are iteratively changed until the difference among measured and predicted label enrichments is reduced.

Ideally, assuming steady state, the distribution of isotopologues would only depend on the distribution of fluxes and the labeled and non-labeled status of the substrates used in the experiment. However, ^13^C propagation from tracer precursors to products is a dynamic phenomenon. Initially, all product metabolites are unlabeled (M+0). Progressively, these products are enriched in ^13^C, with concomitant decrease in M0. Isotopic steady state (Selivanov et al. 2005) is quickly reached for small pools of metabolites but not necessarily for larger pools such as those of fatty acids, glycogen and culture medium metabolites. For these larger pools, M0 values are oversized and may not decrease to the hypothetical value that should be reached at steady state. Accordingly, as an alternative, some software platforms allow for solving the fitting procedure under non isotopic steady state (e.g. Isodyn, INCA among those cited above).

## Pathway analysis

### Enrichment analysis and overrepresentation analysis: the concept

Enrichment analysis as applied in metabolomics is largely based on the approaches implemented for the analysis of transcriptomes, known as Gene Set Enrichment Analysis (GSEA) (Subramanian et al. 2005). The original idea of GSEA is to focus on «gene sets, that is, groups of genes that share common biological function, chromosomal location, or regulation» instead of performing statistics on individual genes. In practice, the goal of the approach is to detect biological processes, such as metabolic pathways, that differ in the experimental dataset of interest versus control datasets.

Replacing gene transcription level with alterations in metabolite concentrations provides a very straightforward approach to interpret metabolomics experiments in terms of changes in the activity of cellular processes. For the application of the GSEA concept in metabolomics, prior information on the biological relationships between metabolites is needed and can be derived from databases of metabolic pathways and reactions (see Table 3 for a list of databases), such as KEGG (Kanehisa et al. 2015, 2017) or MetaCyc (Caspi et al. 2008), or computed based on the similarity of chemical structures (Moreno et al. 2015).

**Table 3:** List of databases of metabolic pathways

Acronym	Full name	Features	Reference
BiGG	Biochemical genetic and genomic knowledgebase of large scale metabolic reconstructions	A genome-scale metabolic reconstruction of the human metabolism	Schellenberger et al. (2010)
BioCyc	BioCyc database collection	A collection of computationally predicted metabolic pathways for nearly 9400 organisms whose genome is available Requires subscription	Caspi et al. (2016)
HumanCyc	Encyclopedia of human genes and metabolism	A partially curated database of metabolic reactions derived from the human genome Requires subscription	Romero et al. (2004)
KEGG	Kyoto encyclopedia of genes and genomes	A collection of manually drawn pathway maps	Kanehisa et al. (2017)
MetaCyc	MetaCyc metabolic pathway database	A curated database of experimentally elucidated pathways	Caspi et al. (2016)
Reactome	NA	A curated, peer-reviewed knowledgebase of biological pathways, including metabolic pathways. It is mainly focused on human pathways	Fabregat et al. (2016)
WikiPathways	NA	A database of biological pathways maintained by and for the scientific community	Kelder et al. (2012)

A related approach is the so called over-representation analysis (ORA, sometimes called annotation enrichment analysis) where one checks whether a group of differentially expressed genes is enriched for a pathway or ontology term by using overlap statistics such as the cumulative hypergeometric distribution (Doniger et al. 2003; Zhong et al. 2004). In contrast with GSEA, ORA does not involve a quantitative assessment of the change in metabolite concentrations. In practice, the application of a hypergeometric test or Fisher’s exact test, with appropriate corrections for multiple testing (e.g. Bonferroni), allows researchers to evaluate whether specific pathways containing metabolites in an experiment-derived list are overrepresented. If the input list contains metabolites featuring different concentrations in different phenotypes (e.g. healthy versus diseased) then the analysis will identify pathways associated with the phenotype changes.

### Metabolite set enrichment analysis (MSEA)

In the application of the GSEA concept to metabolomics, MSEA takes into consideration a quantitative measure associated to each metabolite (e.g. concentration). As the first step of the analysis, metabolites are assigned to specific sets based on one or more reference databases. A group of metabolites are assigned to the same set if they are known to be: (i) involved in the same biological processes (i.e., metabolic pathways, signaling pathways, taken from KEGG) (Kanehisa et al. 2015, 2017); (ii) changed significantly under the same pathological conditions (i.e., various metabolic diseases, taken from the Human Metabolome Database, HMDB) (Wishart et al. 2013) and (iii) present in the same locations such as organs, tissues, or cellular organelles (e.g., also from HMDB).

**Fig. 5:**
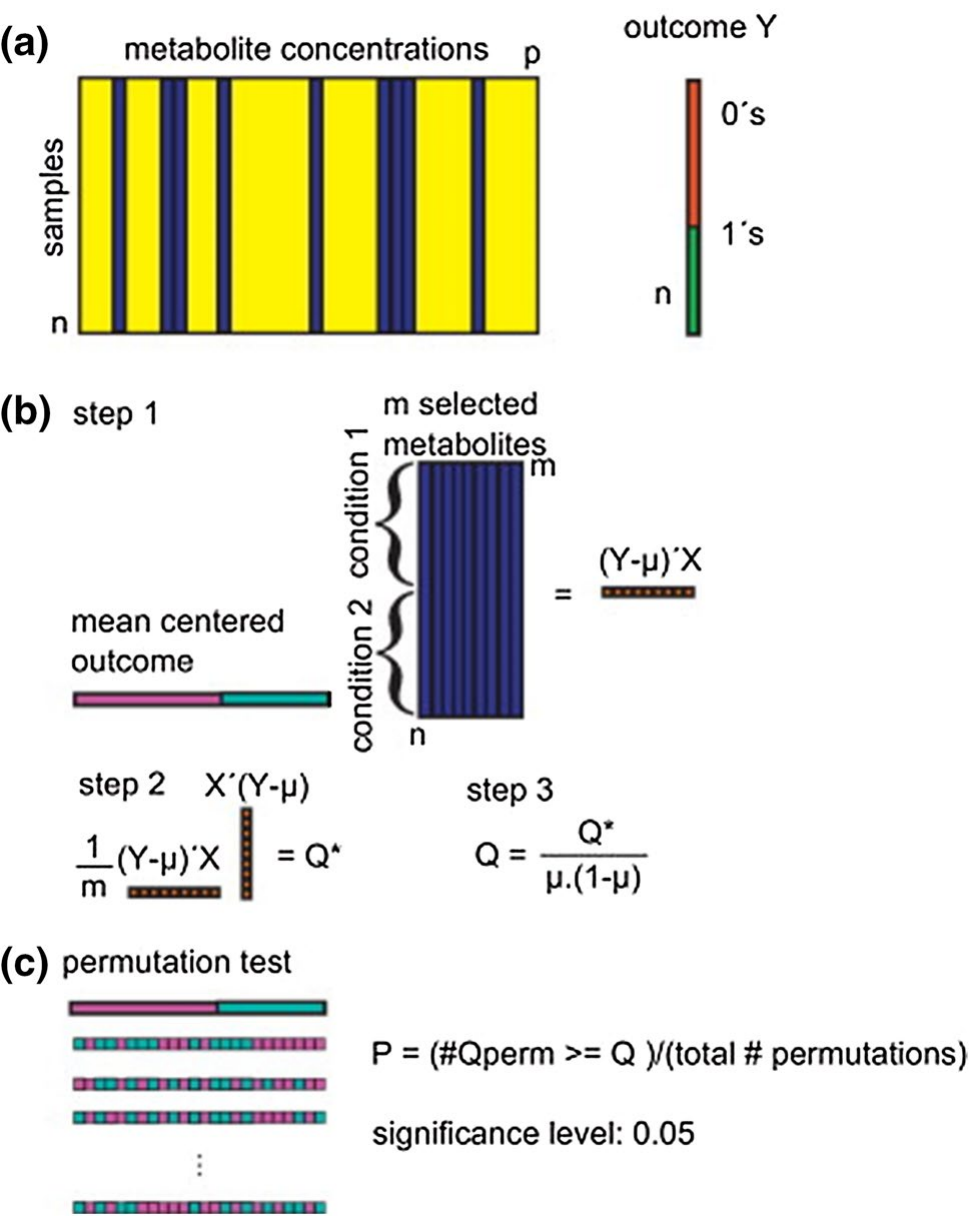
Overview of the Global test. **a** From the autoscaled data matrix, m metabolites belonging to the same pathway are selected. A binary outcome is defined, coded 0 and 1, for instance healthy versus disease. **b** A score statistic **Q** is calculated from the mean centered outcome and the matrix of selected metabolites. **c** The significance of the relation between the group of metabolites (pathway) and the outcome is determined by performing a permutation test. Reproduced with permission from Hendrickx et al. (2012); Copyright (2012) Elsevier B. V

Different strategies exist for performing MSEA depending, among others, on the statistical test applied. In the popular *Globaltest* method (Goeman et al. 2004) *n* samples (e.g. individuals) of *p* metabolites are measured, of which *m* metabolites belonging to the same pathway are selected. The question whether these metabolites behave differently in the two conditions being compared can be translated into the question whether the metabolite levels are predictive for the outcome (Fig. 5). In other words, the question is “does the knowledge of the metabolite concentrations help to improve the prediction of the phenotype (e.g. group, survival, etc…)?” To answer this question, *Globaltest* exploits logistic regression, where the regression coefficients indicate whether a certain metabolite affects the difference between the two conditions. The null hypothesis tested is that no metabolite in the pathway has a different concentration in the two conditions. Thus, the regression coefficients are all zero if the group of selected metabolites has no influence on the phenotype. Unfortunately, the number of coefficients is often much larger than the number of samples leaving no room for classical testing procedures. Goeman et al. (2004) dealt with this issue by assuming that all coefficients belong to a common distribution and demonstrated that the covariance of the distribution is zero under the null hypothesis. Thus, the test becomes whether the covariance is zero (null hypothesis) or different from zero (alternative hypothesis). For this purpose, Rao’s score test (Rao 1948), which is very powerful for detecting small deviations from the null hypothesis, can be applied. The quality parameter that is reported is the *Q*-score statistics, which is based on the differences of metabolite levels between two conditions; a *P*-value is calculated by using permutations. A correction is needed for multiple hypothesis (pathway) testing (e.g. Bonferroni). The *Globaltest* detects consistent differences in patterns of metabolite levels between two conditions. It does not test in which direction a pathway is regulated (up or down), nor it determines how many metabolites have changed concentration levels between two conditions. If the tested pathway is activated or inhibited by the tested condition (e.g. healthy versus diseased patients), the differences in metabolite levels will result in a large *Q*-score and a small *P*-value. However, the results may change, depending on which metabolites are included, i.e. on the completeness of the database(s) from which prior knowledge has been obtained. If the correlation of the missing metabolite(s) with the outcome is almost equal to the average correlation between the outcome and the metabolites included in the pathway, this has almost no effect on the *Q*-score. Instead, if a metabolite that has a much higher or lower correlation to the outcome than average is missing then the *Q*-score will change upon its inclusion. This is an aspect inevitably intrinsic to the MSEA strategy. Databases contain metabolites from only a limited number of pathways, compared to the whole metabolic network of an organism. Consequently, it is possible to test only a relatively small number of pathways and this is an inherent limitation of MSEA.

Another available method is Global Analisys of Covariace (GlobalANCOVA). GlobalANCOVA exploits linear logistic regression and Analysis of Variance (ANOVA) in the framework of a global assessment for a group of metabolites. GlobalANCOVA aims to evaluate the relationship between the metabolite concentrations and the phenotypic covariates. In particular, the aim of GlobalANCOVA is to prove the relevance of certain covariates in explaining the observed metabolite concentration patterns, called covariates of interest. Therefore, two models are compared: the full model (FM), which contains all covariates and the reduced model (RM), which does not have the covariates of interest. The null hypothesis is that both models explain the data equally well. The relevance of the covariates of interest in explaining the observed pattern is proven if the full model explains the observation better than the reduced model. To do so, a squared error is computed for the fitting of the concentration levels of each metabolite. Subsequently, the residual sum of squares (RSS) over all metabolites in the group is computed. Finally, a multivariate test statistic is built based on the RSS values for the full and reduced models (Hummel et al. 2008; Mansmann and Meister 2005; Smyth 2005). The *F*-test is applied to test the null hypothesis and a *P*-value is computed using permutations. A correction for multiple testing is also used. Differently from the Globaltest, GlobalANCOVA evaluates the impact of group membership on the observed metabolite concentration patterns. In other words, GlobalANCOVA practically tests the null hypothesis that the information on the group level does not improve the fitting. The GlobalANCOVA approach allows the inclusion of time-dependent information in a straightforward manner constructed (Hummel et al. 2008).

Hendrickx et al. (2012) first tested the applicability of the Globaltest for metabolomics data and found it effective to highlight the differential behavior of groups of metabolites measured in *E. coli* and *S. cerevisiae* under different environmental conditions.

In a recent study on the impact of sequence variability of mitochondrial DNA on metabolism and ageing, MSEA was used to investigate specific pathways in liver and plasma, showing for example significant changes of glutathione metabolism in both organs (Latorre-Pellicer et al. 2016). MSEA is also useful to assess the impact of therapeutic strategies in disease. For example, the inhibition at an early of glutamine metabolism induces extensive changes in the metabolism of other amino acids but also of the oxidation of branched-chain fatty acids in pancreatic ductal adenocarcinoma cells (Biancur et al. 2017).

### Over representation analysis (ORA)

The most traditional strategy for enrichment analysis in transcriptomics is to take the user’s preselected list of ‘interesting’ genes e.g. genes showing differential expression between two conditions and then iteratively test the enrichment of their annotation terms; Gene Ontology (GO) terms are often used for this purpose. The annotation terms passing the enrichment *P*-value threshold are then reported in a tabular format, usually ordered by the enrichment probability or *P*-value. The calculation of the enrichment *P*-value is related to the number of genes in the list that share the same annotation terms. For example, Gorilla (Eden et al. 2009) enables GO enrichment analysis in ranked lists of genes. Ranking is usually done as a function of expression level or of fold-change in expression. The method identifies, independently for each GO term, the threshold at which the most significant enrichment is obtained. The significance score is corrected for threshold multiple testing. The null assumption is that all configurations of GO term occurrence in the ranked list are equiprobable.

To apply ORA to pathway analysis, the user provides one or more lists of identifiers representing genes/proteins/metabolites significantly associated with the effect of interest. In order to reduce the potential bias when the number of such measured entities is small it is advisable to provide also background lists of all measured genes/proteins/metabolites. Otherwise, all the entities in the predefined pathway database, or in a user-selected sub-ensemble of pathways, are taken into account and used as the background list. Based on the occurrence of its entities within the input lists, the significance of each pathway is assessed by means of a statistical test. ORA analyzes whether, for a given list of metabolites with significantly different concentrations, one particular pathway is overrepresented, i.e. there are more metabolites in the list from that pathway than would be expected by chance. A major difference of ORA with respect to MSEA is that it does not take into account the extent of the fold change of the abundance of metabolites in the list of significant entities: the inclusion of any metabolite in the list typically depends on a fixed arbitrary threshold. In some tools for ORA, however ranked lists are provided, i.e. metabolites are sorted based on the fold-change of their concentration (or their *P*-values). The analysis focuses on whether common terms tend to occur towards the top or the bottom of the list (Kankainen et al. 2011). An application of ORA to patients with mild cognitive impairment (MCI), a transition phase between normal aging and Alzheimer’s disease (AD), showed that the pentose phosphate pathway was differently regulated in MCI patients who later progressed to AD with respect to patients who remained stable (Oresic et al. 2011).

The common weakness of tools performing ORA is that the linear output of terms can be very large and overwhelming (from hundreds to thousands), and this can make difficult to grasp potential interrelationships of relevant terms. In addition, the quality of the pre-selected metabolite lists has a deep influence on the enrichment analysis, making the output unpredictably sensitive to changing statistical methods or cutoff thresholds. In particular, it is inappropriate to use all the metabolites of the metabolite set library as the reference metabolome, because there is no analytical platform that can measure all these metabolites with the same probability. Thus, the choice of the platform rather than the experimental conditions may cause the observed metabolite enrichment. To tackle this problem, the user may upload a platform-specific reference metabolome. This is an option provided, for example, in the implementation available in MetaboAnalyst (Xia et al. 2015). Finally, since multiple hits on a given pathway are required to achieve statistical significance, ORA is of limited usefulness for small-sized pathways like glutathione biosynthesis pathway, which contains only ten compounds.

Due to their intrinsic differences, MSEA and ORA may not give the same results and potentially lead to unlike biological interpretation of the same experimental data. This has been demonstrated for a small set of microarray data, where different GO terms and therefore different biological processes were identified by Globaltest and GOEAST (a web-tool for the analysis of GO term enrichment) (Hulsegge et al. 2009).

### Pathway activity profiling (PAPi)

PAPi allows users to compare the activity of metabolic pathways under different experimental conditions (Aggio et al. 2010). The underlying concept is to associate Activity Scores to each pathway in a set obtained from the KEGG database by averaging the relative abundance of all detected metabolites assigned to that pathway, normalized by a scaling factor that takes into account that not all metabolites are detected. The comparison of Activity Scores under two or more different experimental conditions for the same pathway can pinpoint changes in activity that are statistically significant, as assessed by a two-sample *t*-test or by ANOVA. PAPi can provide information regarding the impact of environmental conditions and stimuli on metabolite uptake and intracellular metabolic overflow. Metabolic pathway activity is directly related to metabolic flux distribution and thus this kind of analysis can tie directly to fluxomics.

## Concluding remarks

The systems biology approach to the interpretation of metabolomics has the potential to unravel the causative mechanisms leading to the observed metabolomics profile. In this way, there is a paradigm shift from the chemometrics framework that makes metabolomics a hypothesis-generating research field to a framework where metabolomics can provide insights into the biological properties of cell and organism functioning. This shift will unlock the potential of metabolomics and related omics disciplines, such as fluxomics and lipidomics, to fully contribute to the advancement of our understanding of health and disease. In this review, we addressed approaches based on association networks and on pathway analysis. These are useful tools to grasp the complexity of metabolomic profiles; however, they are not sufficient to understand fully the intricacies of the metabolism without dedicated experiments.

Many of the methods described here exploit the lessons learned in other, more mature omics, mainly genomics and transcriptomics, e.g. regarding the validation of their theoretical frameworks. As mentioned several times, a major caveat in untargeted metabolomics is the impossibility of measuring all metabolites in the sample, whose consequences are very difficult to predict.
